# A Systematic review of the factors that affect soccer players’ short-passing ability—based on the Loughborough Soccer Passing Test

**DOI:** 10.1186/s13102-024-00880-y

**Published:** 2024-04-26

**Authors:** Bihan Wang, Bin Wan, Shu Chen, Yu Zhang, Xiaorong Bai, Wensheng Xiao, Changfa Tang, Bo Long

**Affiliations:** 1 College of Physical Education, Hunan Normal University, Changsha, 410006 China; 2 School of Physical Education, Huzhou University, Huzhou, 313000 China

**Keywords:** Soccer, Short-passing ability, Influencing factors, Loughborough Soccer Passing Test

## Abstract

**Objective:**

This study synthesizes evidence from the Loughborough Passing Test to evaluate the short-passing ability of soccer players and summarizes the reported variables that affect this ability to provide support for the development and improvement of short-passing abilities in soccer players.

**Methods:**

In this systematic review using the PRISMA guidelines, a comprehensive search was conducted in Web of Science, PubMed, and EBSCOhost from inception to July 2023 to identify relevant articles from the accessible literature. Only studies that used the Loughborough test to assess athletes' short-passing ability were included. The quality of the included studies was independently assessed by two reviewers using the PEDro scale, and two authors independently completed the data extraction.

**Results:**

Based on the type of intervention or influencing factor, ten studies investigated training, nine studies investigated fatigue, nine studies investigated supplement intake, and five studies investigated other factors.

**Conclusion:**

Evidence indicates that fitness training, small-sided games training, and warm-up training have positive effects on athletes' short-passing ability, high-intensity special-position training and water intake have no discernible impact, mental and muscular exhaustion have a significantly negative effect, and the effect of nutritional ergogenic aid intake is not yet clear. Future research should examine more elements that can affect soccer players' short-passing ability.

**Trial registration:**

https://inplasy.com/., identifier: INPLASY20237.

**Supplementary Information:**

The online version contains supplementary material available at 10.1186/s13102-024-00880-y.

## Introduction

Soccer is a game of skills and strategy, and one of the most crucial techniques is short passing [1–3]. A player's ability to make short passes is important for the team to initiate offense and control the pace of the game. Soccer players can more effectively control the game by strategically use their short passing ability. Making multiple quick, short passes in succession can speed up the game, complete the attacking strategy, and increase pressure on the defence of the opposition, which can provide scoring opportunities [4]. According to a study, most goals are preceded by short passes [5].

**Figure 1:**
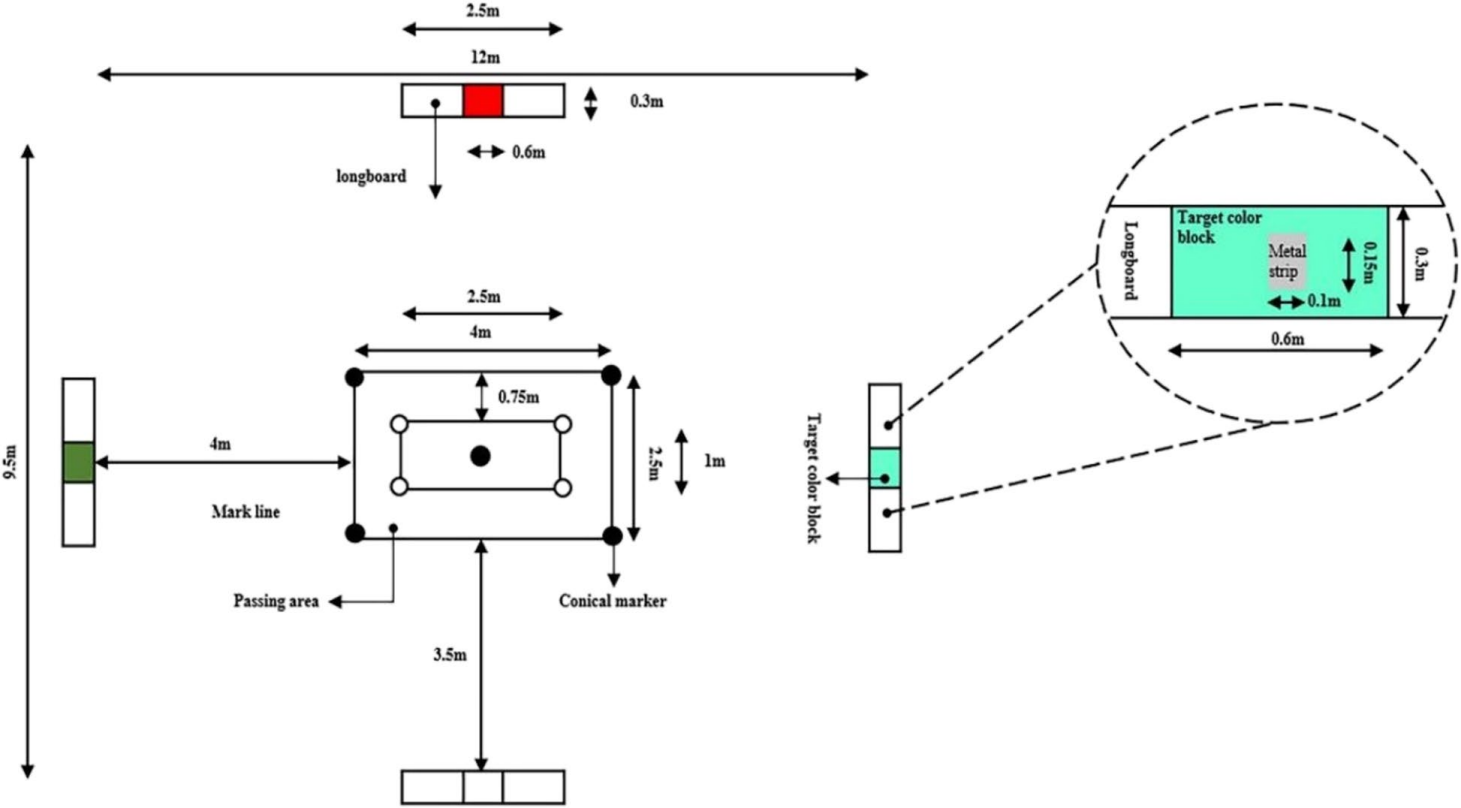
Layout of the LSPT [12, 18]

Players who use short-passing techniques in the game must decide on the pass's timing, strength, and direction under time and space constraints based on the placement of teammates and opponents on the field. However, the conventional short-passing ability assessment employs a single short-passing ability test. The most striking feature of this type of test is that it is performed in a relatively static environment with a short pass to a target or teammate at a known distance and direction; therefore, only motion patterns are shown throughout the test, and it has limited ecological validity [6–9]. This type of test cannot be used to effectively evaluate the short-pass technique of athletes with different levels of competitive ability [10–12]. In contrast to conventional short-passing ability tests, the Loughborough Soccer Passing Test (LSPT), as shown in Fig. 1, as a multitask test, has advantages in the evaluation of athletes' short passes: it requires participants to remember the relative orientation of the target, process oral information for quick decision-making, squelch potential errors, and make flexible cognitive transitions while using their short-passing ability. As a result, the LSPT is consistent with the shifting circumstances of soccer matches [12, 13]. The LSPT procedure is manageable and requires subjects to pass the ball 16 times while surrounded by a rectangular bench. Each bench has a colourful metal strip or coloured cardboard (0.6 × 0.3 m) that can be utilized as a target area to make an effective pass in one of four randomly selected colour sequences. Subjects must complete 16 brief passes of the test as quickly and accurately as they can. Time-related metrics are used to define LSPT scores, including execution time (the amount of time needed to complete 16 passes), penalty time (the amount of time added for mistakes, including incorrect passes and sluggish performance), and total time (execution time plus penalty time). All LSPT time values are inversely correlated with a player's short-passing ability in soccer (a player with a lower LSPT time value has greater short-passing ability). The LSPT is currently used in research on athlete selection in Australia [14], the Netherlands [15], and France [16]. Several studies have shown that the LSPT has good retest reliability and good discriminant validity for players of different sport levels, ages, and genders [12, 17].

Regrettably, despite its importance, there is no systematic review of short-passing abilities or the factors that influence them. Systematic reviews of soccer skills have been conducted on most or all skills or overall athletic performance [19–21], but there is a lack of systematic reviews of specific soccer skills. Due to the importance of short-passing abilities, there is a great need for a more comprehensive analysis of the research on soccer players' short-passing abilities to statistically synthesize the various findings and to examine the factors that affect soccer players' short-passing abilities. The purpose of this paper is to review and analyse research on the factors that affect the short-passing ability of soccer players to contribute to improvements in soccer players’ short-passing ability.

## Methods

This systematic review used the PRISMA guidelines [22] and was registered in the International Platform for Registered Programs for Systematic Reviews and Meta-Analyses (INPLASY); https://inplasy.com, INPLASY202370041.

### Search strategy

A comprehensive, electronic search of the literature was conducted without data restrictions in Web of Science, PubMed, and EBSCOhost on July 10, 2023, using a search strategy developed by two authors (WBH and XWS). The keyword combinations used were: (("Pass" OR "Skill" OR "Technology" OR "Technique" OR "Art" OR "Performance" OR "Ability" OR "Capacity") AND ("Soccer" OR "Football") AND ("LSPT" OR "Loughborough Soccer Passing Test")). Additionally, the researchers explored Google Scholar and the reference lists of the included studies for potential papers that could meet the inclusion criteria for additional related citations.

### Eligibility criteria

The overall, intervention, comparison, outcome, and study design (PICOS) criteria were the inclusion criteria for this study, as detailed in Table 1. Studies were included if they met the following requirements: 1. football players were the subjects of the study; 2. the paper must include at least one study that aimed to assess the effect of a factor or an intervention on the short-passing ability of soccer players; 3. the method used to assess the short-passing ability of the subjects of the study must have been LSPT. Regardless of the factor that influences a soccer player's short-passing ability, any study that met the above three requirements was included in this systematic review.

**Table 1:** Inclusion criteria according to the PICOS conditions

Items	Detailed inclusion criteria
Population	Soccer players (healthy)
Intervention	Any type of intervention
Comparison	Two or more groups and single-group trials
Outcome	Short-passing ability
Study designs	Any experimental designs (e.g., randomized controlled trial, randomized cross-over design, quasi-experimental design)

Studies that met the following criteria were excluded: 1. conferences, overviews, newsletters, book reviews, and studies that were not supported by data and were not analysed statistically; 2. studies that did not quantitatively evaluate the short-passing abilities of the subjects or evaluated them without using the LSPT; 3. studies that did not apply to the vast majority of soccer players, such as the effect of a particular religious practice on the short-passing ability of a soccer player of that religion or the effect of a certain factor on the short-passing ability of a soccer player with a disability.

### Study selection

The following procedure was used to choose the papers. First, prior to importing the studies into EndNote X9 to check for duplication, an experienced librarian assisted with the search strategy by putting key phrases into the three major databases to search for articles. Second, to find pertinent research, two independent reviewers (WBH and XWS) examined the titles and abstracts of all identified papers in accordance with the inclusion and exclusion criteria of the study.

### Data extraction

Two independent reviewers (WBH and XWS) completed the data extraction. Any disputes were explored further. When necessary, a third reviewer (BXR) participated until consensus was reached. The records included (1) the author and year of publication; (2) the study design; (3) participant characteristics, namely, age, sex, and athletic level; (4) the characteristics of the intervention; and (5) the final research outcomes.

### Quality assessment

Two authors (WBH and XWS) independently utilized the PEDro scale, with disagreements resolved by a third rater (BXR). The eligibility criteria in the scale were not included in the total score, as they were related to external validity. The total PEDro score ranges from 0 to 10. The higher the score, the better the methodological quality. A score of 8 to 10 indicates a study of excellent methodological quality, 5 to 7 is considered to indicate good quality, 3 to 4 is considered a study of average quality, and values less than 3 points are considered to indicate fair quality. A score lower than 3 is considered a poor-quality study [23].

## Results

**Figure 2:**
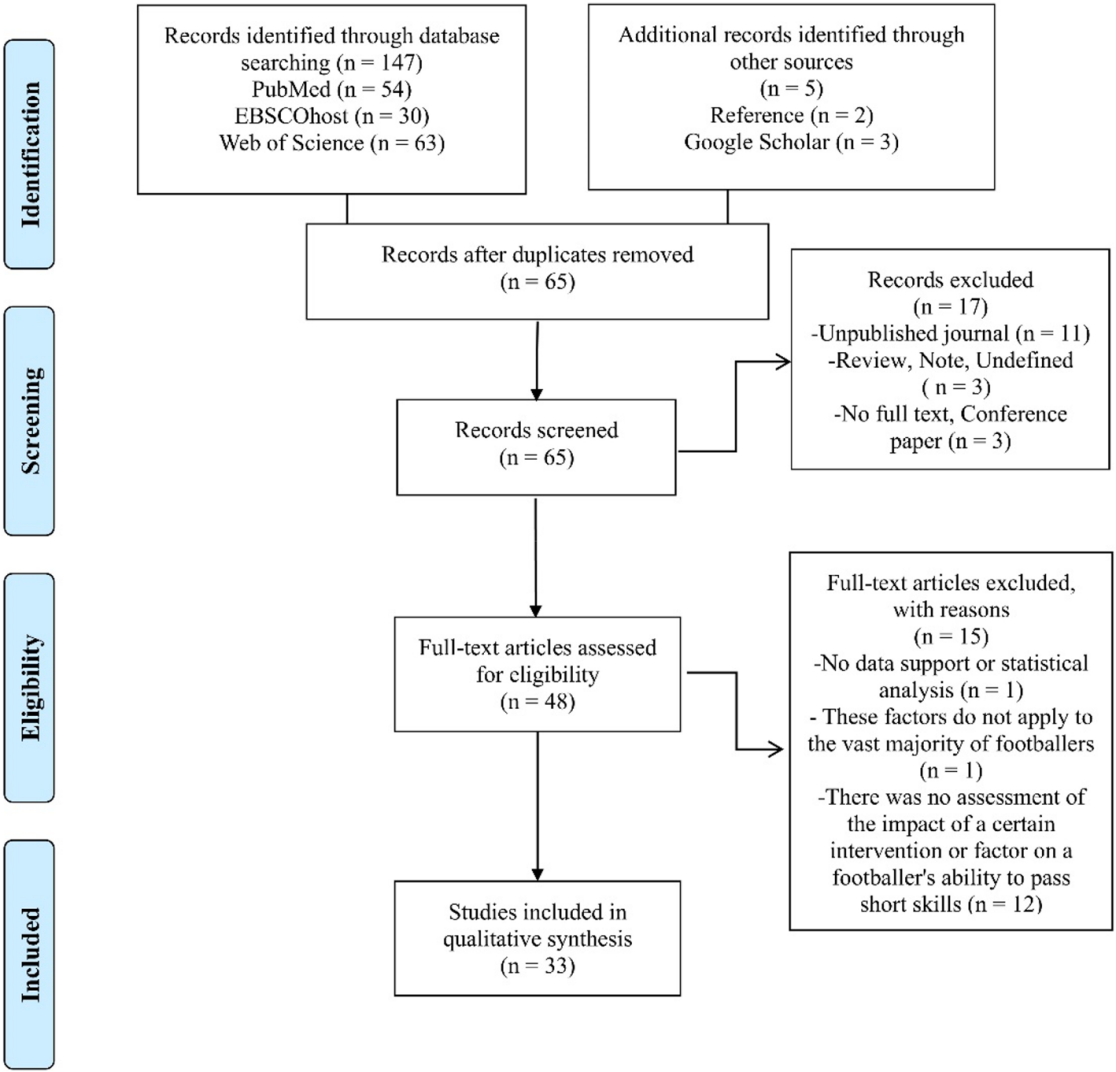
PRISMA flow chart of the study selection process

As shown in Fig. 2, the electronic search of the relevant databases yielded 147 potentially relevant articles (54 from Web of Science, 30 from PubMed, and 63 from EBSCOhost), while an additional five studies were found through Google Scholar and references. The titles and abstracts of 65 publications were evaluated for conformity after duplicates were eliminated (*n* = 87). After 17 items were deleted at the title and abstract levels, the remaining 48 articles were read. Following this reading, an additional 15 publications were excluded, and 33 studies that met all the inclusion criteria for the systematic review were retained. The characteristics of the included studies are detailed in Table 2.

**Table 2:** Characteristics of studies included in this review

Study	Design	Sex/age/athletic level	Intervention (EG/CG)	Outcomes
Training (*n* = 10)				
Fitness training (*n* = 4)				
Impellizzeri et al. [24]	RCT	Male/Age: 17.8 ± 0.6yr/Youth players	Aerobic interval training/Low-intensity technical and tactical training	^2, EG##↓
Zago et al. [25]	Repeated-measures study	Male/ 11.5 ± 0.27yr/ Regional sub-elite players	Technology combined with agility training /Traditional training	^1, EG##↓
Cè et al. [26]	RCT	Male/EG: 10 ± 0.5yr, CG: 10 ± 0.7yr/ Amateur player	Balance training/Mix competition	^1, *EG##↓(very large effect)
Tarakci, Pinar[27]	Non-RCT	Male/EG: 19.03 ± 0.6yr, CG: 18.82 ± 0.65yr/Players with 5 years of game experience	Endurance + Strength training/Strength + Endurance training	EG##↓ CG##↓
Small-sided games training (n = 2)				
Eniseler et al. [28]	RCT	Male/16.9 ±1.1yr/ Professional players	Small-sided games training/Repeat sprint training	EG##↓
Özcan, Şahan [29]	RCT	Male/EG: 18.43 ± 1.47yr, CG: 18.54 ± 1.54yr/ Amateur players	Small-sided games training/Traditional aerobic training	EG##↓
Zois et al. [30]	RCD	Male/23.6 ± 4.1yr/ Professional players	1: Leg press training, 2: Small-sided games training/Passive rest	*EG1##↔ EG2##↓(medium effect) CG##↑(medium effect)
Burcak [31]	NR	Male/20.82 ± 1.41yr/ Non-elite players	Warm-up drills using the 4-ball/Warm-up drills using the 5-ball	#↓
Kaya et al. [32]	Repeated-measures study	Male/22.07 ± 2.87yr/ Professional players	Foam axis rolling training/Passive rest	EG##↔
High-intensity position-specific training (*n* = 1)				
Cuong Le et al. [33]	Single-cohort sequential design	Male/16.1 ± 0.4yr/ Players who have participated in professional football training and national youth competitions	High-intensity position-specific training	EG##↔
Fatigue (*n* = 9)				
Mental fatigue (*n* = 5)				
Smith et al. [34]	RCD	Male/19.6 ± 3.5yr/Professional players	Stroop mission/Leisurely magazine reading	^2，#↑
Smith et al. [35]	RCD	Male/19.6 ± 3.5yr/Professional players	Stroop mission/Leisurely magazine reading	^3，#↑
Greco et al. [36]	RCD	Male/15.0 ± 1.1yr/Youth players	"Brain It On" software/Control state (blank control)	#↑
Filipas et al. [37]	NR	Male/U14, U16, U18/Players competing at national level	U14s (EG1), U16s (EG2) and U18s (EG3) will be on a Stroop mission/U14s (CG1), U16s (CG2), U18s (CG3) relaxing and reading magazine	EG1 vs CG1#↔ EG2 vs CG2#↔ EG3 vs CG3#↑
Bian et al. [12]	RCD	Male/22.0 ± 2.5/Well-trained players	1: LSPT randomized order, 2: LSPT clockwise order, 3: Stroop task	^2，EG1##↑ ^2，EG2##↓ ^2，EG2##↓
Muscle fatigue (*n* = 4)				
Lyons et al. [38]	Repeated-measures study	Male/22.95 ± 5.32yr/College soccer players	1: Moderate-intensity training protocol, 2: High-intensity training regimen /Rest	EG1 vs CG#↓ EG2 vs CG#↑
Rampinini et al. [39]	Quasi-experimental control-period design	Male/17.6 ± 0.5yr/Professional players	1: First half of the match/2: The whole match	EG1##↑ EG2##↑
Draganidis et al. [40]	Repeated-measures study	Male/20 ± 0.7yr/High level competition players	1: Low-intensity resistance exercise program, 2: High-intensity resistance exercise program/Control	EG1##↑ EG2##↑
Lyons et al. [41]	Quantitative cross-sectional design	Female/19.5 ± 3.3yr/Professional elite players and Professional sub-elite players	Complete a high-intensity interval training session Professional elite players/Professional sub-elite players	#↓
Supplement intake (*n* = 9)				
Water intake (*n* = 2)				
Ali et al. [42]	RCD	Female/25.5 ± 5.2yr/Professional players	Water intake/No water intake	#↔
Owen et al. [43]	RCD	Male/22.2 ± 3.1yr/Semi-professional players	1: Intake of water in the amount of sweat loss, 2: Consumption of water at will/No water intake	EG1 vs CG#↔ EG2 vs CG#↔
Nutrient fortification intake (*n* = 7)				
Ali et al. [44]	RCD	Male/21.3 ± 3.0yr/Semi-professional, ex-professional or players who have reached at least college 1st or 2nd team standards	Intake of carbohydrate solution/Placebo intake	#↔
Ali, Williams [45]	RCD	Male/20.9 ± 2.5yr/Semi-professional or non-professional players from college teams	Intake of carbohydrate solution/Placebo intake	#↔
Foskett et al. [46]	RCD	Male/23.8 ± 4.5yr/Regional top league players	Intake of caffeine solution/Placebo intake	#↓
Gant et al. [47]	RCD	Male/21.3 ± 3yr/Class players	Intake of carbohydrate caffeine solution/Intake of carbohydrate solution	#↔
O'Reilly, Wong [48]	RCD	Male/23 ± 2.9yr/College players	Intake of carbohydrate solution/Intake of carbohydrate-free solutions	#↓
Andrade-Souza et al. [49]	RCD	Male/25.4 ± 2.3yr/College players	1: Intake of carbohydrate solution, 2: Intake of caffeine solution, 3: Intake of carbohydrates caffeine solution/Placebo intake	None of the differences between the groups were statistically significant
Shabir et al. [50]	Double-dissociation design	Male/22 ± 5yr/Casual players	1: Placebo intake and informed of placebo, 2: Intake of caffeine solution and informing placebo, 3: Intake of placebo and informed caffeine solution, 4: Intake of caffeine solution and informing about caffeine solution	None of the differences between the groups were statistically significant
Others (*n* = 5)				
Motivation (*n* = 1)				
Barte et al. [51]	2 × 2 design	Male/24.3 ± 4.7yr/Amateur players	Verbal motivation and monetary motivation/Not be motivated	#↓
Verbal interaction (*n* = 1)				
Khalifa et al. [52]	NR	Male and Female/15.4 ± 0.59yr/Amateur student players	Verbal interaction/Not has verbal interaction	EG##↓
Soccer field (*n* = 1)				
O’Meagher et al. [53]	NR	Male/12.7 ± 0.5yr/Top players in the school soccer game	1: Indoor resilient wood flooring, 2: Grasslands, 3: Artificial turf	EG1 vs EG2#↓ EG1 vs EG3#↓ EG2 vs EG3↔
Visual observation (*n* = 1)				
Vansteenkiste et al. [54]	NR	Male/8-10yr/High-level and low-level players in soccer academies	Wearing an eye movement recorder to complete a LSPT Soccer academy high level players/ Soccer academy low level players	#↓
Salbutamol (*n* = 1)				
Halabchi et al. [55]	RCD	Male/17.2 ± 0.8yr/Professional junior soccer players	Intake of salbutamol/Placebo intake	#↔

Note: The evaluation indexes of “outcomes” are all LSPT; in LSPT, there are three indexes: total time, execution time, and penalty time (total time = execution time + punishment time). All the time values of LSPT are inversely proportional to the soccer short-passing skill, i.e., the lower the value of the player's LSPT time is, the better the short-passing skill The default display in the outcomes section is the LSPT total time metric, and in cases where a study does not have a significant difference in the LSPT total time metric (*p* < 0.05) but has a significant difference in the LSPT execution time metric or the penalty time metric, the metrics with significant differences will be reported, and in addition, when a study does not report or use the LSPT total time metric, the outcomes section will report the LSPT execution time metric or the penalty time metric; ^1, LSPT execution time; ^2, LSPT penalty time; ^3, This study statistically analyzes the number of targeting errors when a player performs LSPT based on the rule of LSPT penalty time, so the number of targeting errors when a player performs LSPT is shown here; #↑, LSPT time values were significantly higher in the experimental group post-test than in the control group (or experimental groups 2 and 3) post-test; #↔, no statistically significant between-group difference; #↓, LSPT time values were significantly lower in the experimental group post-test than in the control group (or experimental groups 2 and 3) post-test; ##↑, from pre-test to post-test LSPT time values increased significantly within the group; ##↔, there was no significant change in LSPT time values from pre-test to post-test within the group; ##↓, from pre-test to post-test LSPT time values decreased significantly within the group; *, P-values were not used in the statistical analyses of this study, but rather, effect sizes (ES) were used; *NR*: Not Reported or Not Explicitly Reported; *CG*: control group; EG. experimental group; *yr*: year; RCT: randomized controlled trial; *Non-RCT*: Non-randomized controlled trial; *RCD*: randomized cross-over design

### Demographic characteristics

The pertinent details of the studies are presented in Table 2. The age of the players ranged from 8 to 25.5 ± 5.2 years. With regard to the players’ gender, most of the studies reported male players, two studies examined female players [41, 42], and only one study reported on male and female players [52].

### Intervention characteristics

**Figure 3:**
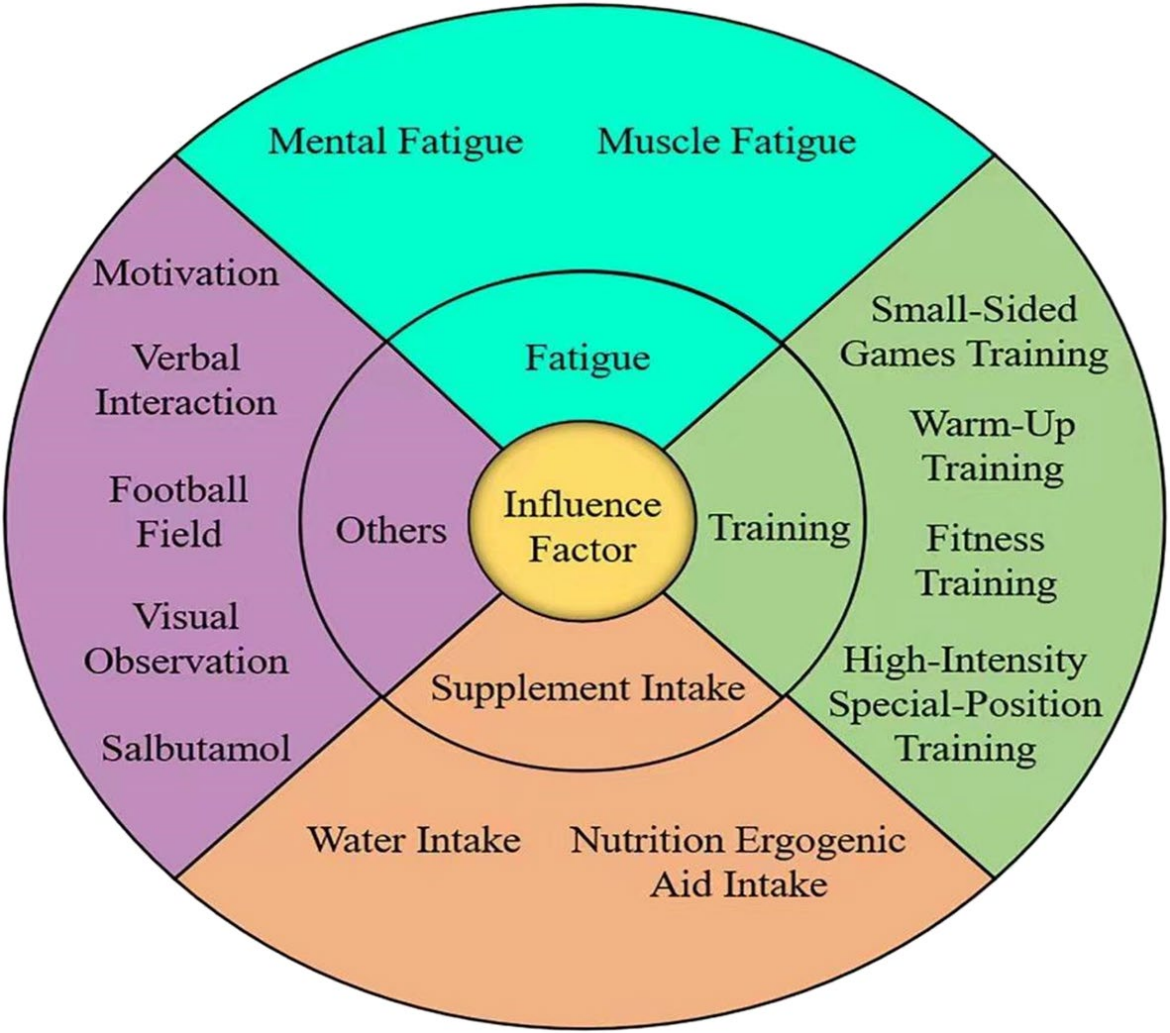
Influence Factor Chart of Short-passing Skill of Soccer Players

For ease of generalization and induction, other factors included motivation, soccer field, verbal interaction, visual observation, and salbutamol intake; these are presented in Fig. 3. Of the 33 included studies, 24 were one-time intervention studies and 9 were long-term intervention studies. Interventions/influencing factors included training (*n* = 10), fatigue (*n* = 9), supplement intake (*n* = 9), and other factors (*n* = 5). The ten papers on the influence of training on football players' short-passing ability included fitness training (*n* = 4), small-sided games training (*n* = 2), warm-up training (*n* = 3) and high-intensity position-specific training (*n* = 1). The 9 papers on the effect of fatigue on short-passing ability in soccer players included mental fatigue (*n* = 5) and muscle fatigue (*n* = 4). The 9 papers on the effect of supplement intake on short-passing ability in soccer players included water intake (*n* = 2) and nutritional ergogenic aid intake (*n* = 7). The five papers on other factors that influence football players' short-passing ability included motivation (*n* = 1), verbal interaction (*n* = 1), football field (*n* = 1), visual observation (*n* = 1) and salbutamol intake (*n* = 1).

Among the studies (*n* = 10) on the effects of training on short-passing ability in soccer players, with the exception of two one-time intervention studies [30, 32], 1) all studies explicitly reported the total duration of the intervention, with the shortest being 5 days [31] and the longest being 22 weeks [25]; 2) most of the studies explicitly reported the duration of each intervention, with the shortest being 16 minutes [33] and the longest being 98 minutes [25] and only two studies failing to explicitly report the duration of each intervention [27, 31]; 3) all studies explicitly reported the frequency of intervention, which was once a day in one study [31], twice a week in three studies [28, 29, 33], three times a week in two studies [26, 27], between 2 times a week and 4 times a week in one study [25], and 2 times in the first week and 3 times in weeks 2 to 4 in one study [24]. In the studies in which fatigue affected the short-passing ability of soccer players (*n* = 9), all reported in detail the intervention protocols used. In terms of mental fatigue, four studies used the Stroop task [18, 34, 35, 37], one study used Brain It On software [36], one study used the LSPT random order and clockwise order tasks in addition to the Stroop task [18], and four studies performed muscle training [38], soccer matches [39], high-intensity interval training [40], and resistance training [41]. In studies on the effects of supplement intake on short-passing ability in soccer players (*n* = 9), 1) all studies reported the intake dose of supplements; 2) all studies explicitly reported the type of supplement ingested, including water, carbohydrate solution, caffeine solution, and carbohydrate caffeine solution (i.e., carbohydrate solution mixed with caffeine solution). In two studies only water was ingested [42, 43], in three studies only carbohydrate solutions were ingested [44, 45, 48], two studies used only caffeine solution [46, 50], carbohydrate solutions and carbohydrate caffeine solutions were ingested in one study [47], and carbohydrate solution, caffeine solution, and carbohydrate caffeine solution were ingested in one study [49]. All other studies of factors that affect short-passing ability in soccer players (*n* = 5) provided a clear description or explicit definition of the substance or method of intervention.

### Study quality assessment

The quality of the studies is presented in Table 3. The PEDro checklist was used to assess the quality of the included studies. The results showed that eight studies received a score of 3 or 4, indicating average quality, and 18 studies scored 5 to 7 points, which was considered good quality. Moreover, seven studies had scores ranging from 8 to 10 points and were considered to have excellent methodological quality.

**Table 3:** Summary of methodological quality assessment scores

References	Impellizzeri et al. (2008) [24]	Zago et al. (2016) [25]	Cè et al. (2018) [26]	Tarakci, Pinar (2021) [27]	Eniseler et al. (2017) [28]	Özcan, Şahan (2018) [29]	Zois et al. (2013) [30]	Burcak (2015) [31]	Kaya et al. (2021) [32]	Cuong Le et al. (2023) [33]	Smith et al. (2016) [34]
Eligibility criteria	1	1	1	1	1	1	1	0	1	1	1
Random allocation	1	1	1	0	1	1	1	0	1	0	1
Allocation Concealment	1	1	1	0	1	1	1	0	1	0	1
Baseline Comparability	1	1	1	1	1	1	1	1	1	1	1
Blind Participants	0	0	0	0	0	0	0	0	0	0	1
Blind Therapist	0	0	0	0	0	0	0	0	0	0	0
Blind Assessor	0	0	0	0	0	0	0	0	0	0	1
Follow-up	1	0	0	0	0	0	0	0	0	0	0
Intention to Treat Analysis	1	1	1	1	1	1	1	1	1	1	1
Between Group Comparisons	1	1	1	1	1	1	1	1	1	0	1
Point Measure and Variability	1	1	1	1	1	1	1	1	1	1	1
Total PEDro Score	7	6	6	4	6	6	6	4	6	3	8
References	Smith et al. (2017) [35]	Greco et al. (2017) [36]	Filipas et al. (2021) [37]	Bian et al. (2022) [18]	Lyons et al. (2006) [38]	Rampinini et al. (2008) [39]	Draganidis et al. (2013) [40]	Lyons et al. (2021) [41]	Ali et al. (2011) [42]	Owen et al. (2013) [43]	Ali et al. (2007) [44]
Eligibility criteria	1	1	1	1	1	1	1	1	1	1	1
Random allocation	1	1	1	1	0	0	1	0	1	1	1
Allocation Concealment	1	1	1	1	0	0	1	0	1	1	1
Baseline Comparability	1	1	0	1	1	1	1	0	1	1	1
Blind Participants	1	0	1	0	0	0	0	0	0	0	0
Blind Therapist	0	0	0	0	0	0	0	0	0	0	0
Blind Assessor	0	0	1	0	0	0	0	0	0	0	0
Follow-up	0	0	0	0	0	0	0	0	0	0	0
Intention to Treat Analysis	1	1	1	1	1	1	1	1	1	1	1
Between Group Comparisons	1	1	1	1	1	1	1	1	1	1	1
Point Measure and Variability	1	1	1	1	1	1	1	1	1	1	1
Total PEDro Score	7	6	7	6	4	4	6	3	6	6	6
References	Ali, William (2009) [45]	Foskett et al. (2009) [46]	Gant et al. (2010) [47]	O'Reilly, Wong (2013) [48]	Andrade-Souza et al. (2015) [49]	Shabir et al. (2019) [50]	Barte et al. (2019) [51]	Khalifa et al. (2020) [52]	O’Meagher et al. (2022) [53]	Vansteenkiste et al. (2022) [51]	Halabchi et al. (2017) [55]
Eligibility criteria	1	1	1	1	1	1	1	1	1	1	1
Random allocation	1	1	1	1	1	1	1	1	0	0	1
Allocation Concealment	1	1	1	1	1	1	1	1	0	0	1
Baseline Comparability	1	1	1	1	1	1	0	1	1	0	1
Blind Participants	1	1	1	0	1	1	0	0	0	0	1
Blind Therapist	1	1	1	0	1	1	0	0	0	0	1
Blind Assessor	0	0	0	0	0	0	0	0	0	0	0
Follow-up	1	0	0	0	0	0	0	0	0	0	0
Intention to Treat Analysis	1	1	1	1	1	1	1	1	1	1	1
Between Group Comparisons	1	1	1	1	1	1	1	1	1	1	1
Point Measure and Variability	1	1	1	1	1	1	1	1	1	1	1
Total PEDro Score	9	8	8	6	8	8	5	6	4	3	8

### Outcome and measures

The results of the current study were divided into groups based on the various interventions and influencing factors that were found to have an impact on soccer players' short-passing ability.

#### The effect of training on the short-passing ability of soccer players

##### Fitness training

Four studies examined the impact of fitness training on soccer players' short-passing abilities [24–27]. The fitness training methods included aerobic interval training [24], skill combined with agility training [25], balance training [26], and strength combined with endurance training [27]. The subjects included amateur players [26], youth players [24], players with five years of experience [27], and regional sub-elite players [25]. The results of these studies demonstrate that fitness training improves soccer players' short-passing abilities and is more effective than the training methods used in the control groups of the respective studies.

##### Small-sided games training

This review comprises two studies that examined the impact of small-sided games training on soccer players' short-passing abilities [28, 29]. The participants included amateur players [29] and professional players [28]. Both studies found that small-field match training improved short-passing ability in soccer players and demonstrated that small-field match training was more effective than repetitive sprint training and conventional aerobic interval training, respectively, which were used by their control groups.

##### Warm-up training

The influence of warm-up training on soccer players' short-passing abilities was examined in three studies [30–32]. One of these studies examined pre-match warm-up training, while the other two explored halftime rewarm-up training. These studies used four warm-up training methods, including passing warm-up training [30], foam axle rolling training [32], leg press training, and small-sided games training [30]. The participants included non-elite players [31] and professional players [30, 32]. Of the four training methods, foam axle rolling training [32] and leg press training [30] performed during halftime did not significantly affect players’ short-passing ability, while the remaining two warm-up training methods positively affected players’ short-passing ability [30, 31].

##### High-intensity special-position training

Only one study included in this systematic review presented inferences about the effect of high-intensity special position training on soccer players' short-passing abilities [33]. The participants in this study were national youth events and professional soccer training services. This study revealed no improvement in short-passing ability after high-intensity special-position training [33].

#### The effect of fatigue on the short-passing ability of soccer players

##### Mental fatigue

This review included five studies that examined the impact of mental fatigue on soccer players' short-passing abilities [18, 34–37]. The participants included youth players [36], trained players [18], players competing at the national level [37], and professional players [34, 35]. These five studies revealed a significant negative impact of mental weariness on soccer players' short-passing abilities.

##### Muscle fatigue

This systematic review comprised four studies that examined how soccer players' short-passing abilities were affected by muscular exhaustion [38–41]. Importantly, two of the studies provided indirect confirmation rather than directly investigating how muscular exhaustion affects soccer players' short-passing abilities [40, 41]. The participants included college soccer players [38], high-level competition players [40], professional elites, sub-elite players [41], and professional footballers [39]. These four studies demonstrated that muscle exhaustion can significantly impair soccer players' short-passing abilities.

#### The effect of supplement intake on short-passing ability in soccer players

##### Water intake

This review included two trials that examined the impact of water intake on soccer players' short-passing abilities [42, 43]. The participants included semi-professional players [43] and professional players [42]. The intake of water had no discernible impact on players' ability to produce short passes in both experiments.

##### Nutrition ergogenic aid intake

This review comprised seven trials to confirm the impact of nutrition ergogenic aid intake use on football players' short passing ability [44–50]. The subjects included semi-professional players, ex-professional players or players who had reached at least college 1st/2nd team standards [44], semi-professional or non-professional players from college teams [45], regional top league players [46], class players [47], college players [48, 49], and casual players [50]. Only two studies reported a significant positive effect on players' short-passing ability when they ingested a carbohydrate solution [48] or a caffeine solution [46]. The results of the remaining five studies indicated that the ingestion of a carbohydrate solution, a caffeine solution, or a carbohydrate caffeine solution did not have a significant effect on players' short-passing ability [44, 45, 47, 49, 50].

#### The effect of supplement intake on short-passing ability in soccer players

This review included five studies that examined additional variables that influenced soccer players' short-passing abilities [51–55]. The participants included amateur players [51], amateur student players [52], top players in school soccer games [53], soccer academy high-level players, soccer academy low-level players [54] and professional junior soccer players [55]. In five studies, motivation [51] and verbal interaction [52] were reported to positively influence players' short-passing ability. O’Meagher et al. (2022) reported no significant difference in players' short-passing ability between grass and artificial turf. One study reported the important effect of visual observation on players' short-passing ability [54]. Another study showed that salbutamol intake did not have a significant effect on players' short-passing ability [55].

## Discussion

### The effect of training on the short-passing ability of soccer players

#### Fitness training

The growth and performance of soccer players’ technical and tactical skills depend on their level of fitness. The four studies that examined how short-passing abilities in soccer players were affected by fitness training all concluded that players could benefit from the training techniques used in their studies, which included aerobic interval training [24], strength and endurance training [27], skill and agility training [25], and balance training [26]. This means that a player's short-passing ability benefits not only from technical training but also from fitness training. The training techniques employed in these studies involve only a portion of the fitness training approach, including endurance training, balance training, and strength training. Some studies support the findings of earlier research that showed that fitness training can enhance athletes' abilities [56–58]. In fact, the same rationale that supports the positive effects of fitness training on athletes' specialized skills in other sports likely applies to the short-passing abilities of soccer players. In addition to athletes’ mastery of the technique itself, athletes’ physical attributes are crucial to the use of the skill. For instance, an athlete's balance directly influences the mass of the short passing, which is a dynamic unilateral technical movement [59, 60], especially when a game-time physical altercation with the opponent occurs. Future studies should examine the effects of various fitness training programmes on soccer players' short-passing abilities.

#### Small-sided games training

Soccer training for small-sided games is referred to as skill-based match training [61] or match-based training [62] and is typically played on a smaller pitch. According to the two included studies on the impact of small-sided games training on soccer players' short-passing ability [28, 29], small-sided games training considerably enhances players' short-passing ability. Small-sided games training simulates the athletic demands, physiological intensity, and technical requirements of a soccer game. Compared with traditional short-passing practice (e.g., one-on-one passing, multiple passes to each other), small-sided games training forces players to use short passes more frequently under increased defensive pressure and reduced field size due to the limitations of the rules. In other words, small-sided games training allows players more opportunities to use and practice short passes under time and space pressure [62, 63]. This may also explain why small-sided games training improves soccer players' short-passing ability more significantly than traditional short-passing training or other training methods. This means that coaches and players can use small-sided games training drills to improve short passes in real scenarios that are more similar to games.

#### Warm-up training

Soccer training before a game is essential. In recent years, researchers have examined various warm-up training strategies, such as rewarming up during the game's halftime break and conventional pregame warm-up training, as the methods and means of warm-up training have become more varied. In comparison to a ball size of five, Burcak's (2015) study found that pre-match warm-up training with a ball size of four had a positive effect on players' short-passing ability. In a randomized crossover experiment, Zois et al. (2013) discovered that practising for a small-sided game during halftime increased players' short-passing ability. In contrast, the halftime leg press drill had little impact on players' short-passing ability. Similarly, randomized crossover research by Kaya et al. (2021) revealed that halftime foam-axis rolling drills had no positive impact on players' short-passing ability [64, 65]. Nevertheless, due to the lower intensity, foam-axis rolling training and leg press training during halftime tend to reduce muscle temperature in athletes who have recently concluded a game's first half. Based on these findings, athletes may decide to maintain their muscular temperature by engaging in rewarming exercises during halftime. However, it is crucial to remember that each player must be evaluated individually. If a player is extremely exhausted at halftime, rewarming up for training may worsen his or her short passing ability and athletic performance.

#### High-intensity special-position training

Soccer players who engage in high-intensity position-specific training practise the skill most pertinent to their position at a high level (90% HRmax) [33]. Compared to the impact of small-sided games training on soccer players' short-passing abilities, this produces the opposite outcome. Due to the limitations of the field size, small-sided games training may offer more possibilities for practising short-passing techniques. High-intensity position-specific training, in contrast, includes many additional elements and requires less time to improve short-passing ability. This indicates that high-intensity special-position training is used by coaches and players to enhance short passing, which is an unwise choice.

### The effect of fatigue on the short-passing ability of soccer players

#### Mental fatigue

According to one definition, mental tiredness is a psychobiological condition marked by feelings of exhaustion that can occur during or after prolonged periods of perceived exertion [66, 67]. The five studies in this paper on the effect of psychological exhaustion on soccer players' short-passing abilities all concluded that psychological exhaustion may be detrimental to these abilities [18, 34–37]. This suggests that coaches and players should pay increased attention to this easily overlooked factor that affects short-passing ability. According to Filipas et al. (2021), mental fatigue has a significant negative impact on U18 players' short-passing abilities as well as a negative, albeit nonsignificant, impact on U14 and U16 players' short-passing abilities; total LSPT times are 7.4% (U14) and 4.2% (U16) greater than the control group. Smith et al. (2017) also performed more thorough statistical analyses using the LSPT penalty time rule. In contrast to players who are not mentally weary, mentally fatigued players targeted errors substantially more and completed passes significantly less frequently. These findings confirm earlier studies suggesting that mental weariness impairs athletes' performance [66, 68, 69]. According to some research, mental weariness can impair a player's ability to concentrate, lengthen reaction times for cognitive activities, and increase a player's risk of making mistakes when using short-passing techniques [70]. When using short-passing abilities in soccer, players must maintain a high degree of focus and accurate perception to allow them to make the right choices in a highly dynamic environment and under time and space pressures. As a result, athletes should try to prevent developing premature mental tiredness. Cognitive tasks that require considerable energy typically lead to mental weariness [71]. Therefore, to avoid premature mental fatigue, players should be wary of high levels of pregame cognitive demands (e.g., excessive use of cell phones, tablets, and video games, as well as prolonged cognitive skill training).

#### Muscle fatigue

Short inter-match recovery times (halftime) and high neuromuscular demands during soccer matches may result in muscle fatigue during the game, decreasing players' abilities and fitness, which may have an impact on match performance [72]. Researchers of soccer have paid close attention to the impact of muscular fatigue on short-passing ability, one of the skills most often employed by players in games. Two studies directly reported significant negative effects of muscle fatigue on soccer players' short-passing ability [38, 39], and two other studies provided indirect support that short-passing ability can have significant negative effects on soccer players. After high-intensity interval training, Draganidis et al. (2013) reported that professional sub-elite players' short-passing abilities deteriorated, and Lyons et al. (2021) found that high-level players' short-passing abilities deteriorated. These findings provide circumstantial evidence that players with short-passing abilities can suffer from muscle exhaustion resulting from persistent dynamic exercise and resistance training [73, 74].

These results are consistent with those from earlier investigations. In fact, numerous studies have documented losses in athletic ability and performance that occur as players approach a state of muscular tiredness [75, 76], and one study reported that after fatigue training, a considerable drop occurred in shooting scores [77]. Soccer players’ short-passing abilities might suffer from muscle exhaustion, perhaps as a result of a reduction in muscle functioning capacity [78, 79], which decreases the stability and accuracy of a player's passes. The decrease in players' short-passing ability caused by completing short bursts of high-intensity activity at the same absolute workload is also related to players' physical quality [39]. Therefore, in actual daily training, to prevent premature muscle fatigue from impairing short-passing ability in play, players should enhance their physical training and practice.

### The effect of supplement intake on the short-passing ability of soccer players

#### Water intake

During a game, soccer players exert both mental and physical effort. Under extreme physical and mental strain, the body is susceptible to water loss and mental exhaustion. Reduced endurance and cognitive function can result from dehydration in athletes with up to 2% body weight loss during exercise [80, 81]. Two variables may cause a player's ability to pass short passes to gradually deteriorate throughout sports: dehydration and inadequate water intake. To keep players' short-passing ability or slow down its decline, several studies have tried feeding them a specific volume of water. However, neither of the experiments presented in this study revealed a significant impact of water intake on players' short-passing abilities [42, 43]. These findings suggest that soccer players cannot rely on drinking water during a game to prevent a decrease in short-passing ability.

#### Nutritional ergogenic aid intake

The nutritional ergogenic aid intake is anything that enhances athletic performance. It can be a nutrient, a nutrient metabolite, a food extract (from a plant), or something that is typically present in other items (i.e., caffeine or carbohydrates) [82]. Carbohydrate solution, caffeine solution, and carbohydrate and caffeine solution were utilized in the seven studies that examined the impact of the nutritional ergogenic aid intake on soccer players' short-passing abilities. O'Reilly et al. (2013) and Foskett et al. (2009) reported that ingesting a carbohydrate solution or a caffeine solution significantly improved players' short-passing ability. The other five studies found no evidence that ingesting a carbohydrate solution, a caffeine solution, or a carbohydrate and caffeine solution significantly improved players' short-passing abilities [44, 45, 47, 49, 50]. Three studies indicate that players' short pass ability is positively impacted by nutritional ergogenic supplement intake, but these findings also indicate that this relationship is not statistically significant [44, 45, 50]. Therefore, the impact of nutritional ergogenic aid intake on soccer players' short-passing abilities is unclear and requires additional explanation. In fact, the effects of nutritional ergogenic aid intake on athletes' skills have been similarly ambiguous in other investigations. For instance, Stuart et al. (2005) [83] reported that rugby players who swallowed a caffeine solution had a 10% increase in passing accuracy on the exam. However, rugby players took the same dose of caffeine solution in the study by Assi and Bottoms (2014), and the findings revealed no appreciable impact on test-passing accuracy [84]. Belenky et al. (2005) [85] claimed that ingesting a caffeinated solution enhanced shooting ability, although other studies have demonstrated that doing so did not significantly enhance this ability [86, 87]. Therefore, football players are advised to not employ nutritional ergogenic aid intake to maintain their short-passing abilities or to halt their decline. Future research should confirm these findings with additional randomized, double-blind crossover experiments. Future research is necessary to determine the potential impact of other commonly used nutritional ergogenic aids, such as creatine, L-carnitine, protein, and amino acid supplements, on football players' short pass ability.

### Other factors affecting short-passing ability in soccer players

The five studies that were evaluated in this section of the paper examined five underappreciated or overlooked factors that may affect soccer players' short-passing abilities [51–55]. Barte et al. (2019) reported that various methods of motivating worn-out players while at rest improve athletes' short-passing abilities. This suggests that motivating players makes practical sense for improving short passes, which provides support for coaches who are accustomed to motivating players. Khalifa et al. (2020) suggested that talking to teammates during halftime can improve short-passing abilities (10.2% reduction in overall LSPT time) and can outperform passive rest (4.2% reduction in total LSPT time).

Players' short-passing abilities did not differ significantly between grass and artificial turf, according to research by Meagher et al. (2022) Players should therefore not worry about the effect that being on two different types of turf may have on their short-passing ability. However, indoor springy wood flooring considerably improved players' short-passing abilities over grass and synthetic turf. Nevertheless, indoor 5-a-side soccer games are usually the only tournaments played on indoor resilient wood floors. Visual observation, as shown by Vansteenkiste et al. (2022), has a significant impact on players' short-passing ability. When players utilize short-passing abilities, spending too much time focusing on the ball might prevent them from seeing teammates' and defenders' locations, which can cause them to miss the ideal opportunity to pass the ball or lose possession. This suggests that players should be more observant of the ever-changing conditions on the field rather than just staring at the ball in the ratios. Coaches must be aware of this key point, which can be easily overlooked, and remind players of it during training, and players must recognize it themselves. Additionally, a study revealed that taking salbutamol had no discernible impact on players' short-passing abilities [55].

## Limitations

This study systematically evaluated the factors that affect soccer players' short-passing ability. The results showed that these factors can be divided into positive and negative categories. This study provides a reference and support for soccer coaches and players to improve their short-passing abilities. However, there are a few limitations to this review. 1) Papers in languages other than English were excluded from the study, which influenced the selection of papers. 2) Because it is unknown whether the participants' sex, age, and level of sport affected the intervention effects of some research, the pertinent conclusions should not be extended without due care. 3) Despite the advantages of the LSPT over the conventional short-passing ability test, the LSPT cannot accurately imitate the intricacies of soccer players' use of the short-passing technique in games. 4) The present results should be applied with caution due to the lack of research on some of the influencing elements included in this study, which could affect the accuracy of some of the conclusions. Nevertheless, we believe that the current study can aid in the development and improvement of short-passing abilities in soccer because it examines some relevant strategies and elements.

## Conclusions

This study's findings indicate that a variety of factors can influence soccer players' short-passing abilities. For example, in terms of the effect of training on football players' short-passing abilities, fitness training, small-sided games training, and some warm-up training positively impact these abilities, while high-intensity special-position training has no discernible impact. Mental and muscular exhaustion have a significantly negative effect. In terms of the effect of supplemental intake on football players’ short-passing ability, water intake has no significant effect, and the effect of nutritional ergogenic aid intake is not yet clear. Based on these findings, additional research is encouraged to investigate techniques or variables that affect short-passing ability in soccer players, such as additional training methods (e.g., Specialized short-passing ability training and functional training) and players' own factors (e.g., sleep and mood). However, whether the results of this study apply to all soccer players of all ages, sexes, and athletic levels is unknown. Future research should focus on determining whether a specific subset of the findings is appropriate for a particular group of soccer players. In addition, this study offers only a general directional reference for the sustainable development and improvement of soccer players' short-passing ability.

### Supplementary Information

**Additional file 1.** The original contributions presented in the study are included in the article/supplementary material, further inquiries can be directed to the corresponding authors.

## Data Availability

Data are available on request to the corresponding author by e-mail (03218@zjhu.edu.cn OR changfatang@hunnu.edu.cn OR longbo0811@163.com), and registered in the International Platform for Registered Programs for Systematic Reviews and Meta-Analyses (INPLASY); https://inplasy.com, INPLASY202370041.
